# Biomechanics of footwear design

**DOI:** 10.1186/1757-1146-5-S1-I1

**Published:** 2012-04-10

**Authors:** Richard Smith, Caleb Wegener, Andy Greene, Angus Chard, Alycia Fong Yan

**Affiliations:** 1 Exercise Health and Performance Research Group, The University of Sydney, Sydney, NSW 2041, Australia

## Background

The aim of the workshop is to explore the effects of footwear design on lower limb motion and discuss research findings relevant to the clinic and physical activity. Delegates will be actively involved in a motion capture process where important lower limb joint stress variables will be displayed in real time. The University of Sydney researchers will raise footwear issues from a range of areas such as children physical activity, adult walking and running, specific footwear such as thongs (flip-flops), dance and experimental methodology.

During or on completion of this workshop, participants will be able to:

• Critically analyse footwear characteristics that are likely to influence lower limb function during physical activity.

• Experience the use of state of the art technology to analyse lower limb mechanics.

• Discuss the models and methods used to build knowledge about the effect of footwear on lower limb mechanics.

• Recount clinical and research outcomes for the effect of footwear on lower limb biomechanics during physical activity.

• Apply biomechanical principles to the effective choice of footwear for a range of physical activities.

